# Extracellular RNA in oncogenesis, metastasis and drug resistance

**DOI:** 10.1080/15476286.2024.2385607

**Published:** 2024-08-06

**Authors:** Hannah Nelson, Sherman Qu, Jeffrey L. Franklin, Qi Liu, Heather H. Pua, Kasey C. Vickers, Alissa M. Weaver, Robert J. Coffey, James G. Patton

**Affiliations:** a Department of Biological Sciences, Vanderbilt University and Vanderbilt University Medical Center, Nashville, TN, USA; b Center for Extracellular Vesicle Research, Vanderbilt University School of Medicine, Nashville, TN, USA; c Department of Cell and Developmental Biology, Vanderbilt University School of Medicine, Nashville, TN, USA; d Department of Biostatistics, Vanderbilt University and Vanderbilt University Medical Center, Nashville, TN, USA; e Department of Pathology, Microbiology, and Immunology, Vanderbilt University and Vanderbilt University Medical Center, Nashville, TN, USA; f Department of Medicine, Vanderbilt University and Vanderbilt University Medical Center, Nashville, TN, USA

**Keywords:** extracellular vesicles, nanoparticles, RNA, Cancer, Tumor microenviroment, metastasis, drug resistance

## Abstract

Extracellular vesicles and nanoparticles (EVPs) are now recognized as a novel form of cell–cell communication. All cells release a wide array of heterogeneous EVPs with distinct protein, lipid, and RNA content, dependent on the pathophysiological state of the donor cell. The overall cargo content in EVPs is not equivalent to cellular levels, implying a regulated pathway for selection and export. In cancer, release and uptake of EVPs within the tumour microenvironment can influence growth, proliferation, invasiveness, and immune evasion. Secreted EVPs can also have distant, systemic effects that can promote metastasis. Here, we review current knowledge of EVP biogenesis and cargo selection with a focus on the role that extracellular RNA plays in oncogenesis and metastasis. Almost all subtypes of RNA have been identified in EVPs, with miRNAs being the best characterized. We review the roles of specific miRNAs that have been detected in EVPs and that play a role in oncogenesis and metastasis.

## Introduction

**Figure 1.**
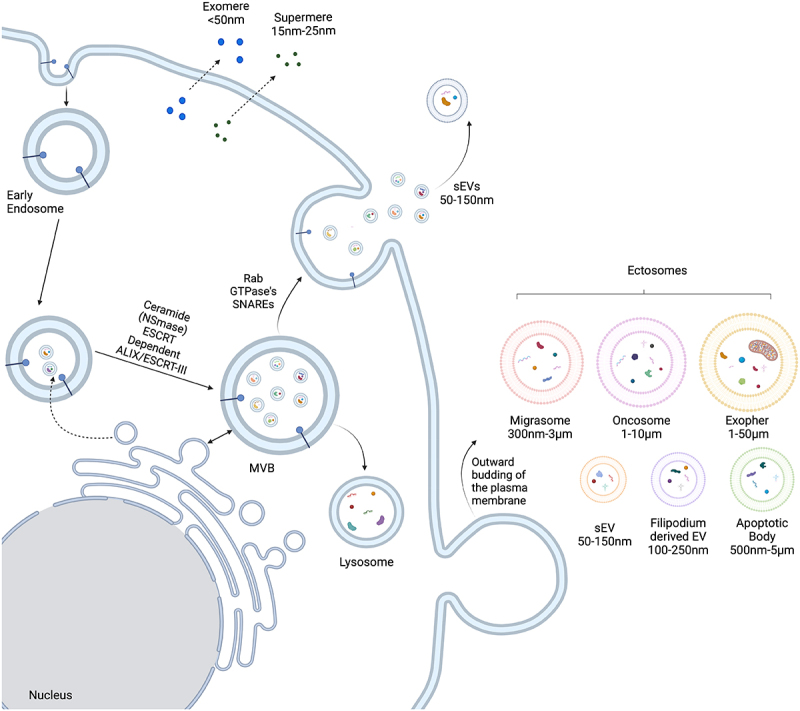
Biogenesis of extracellular vesicles.

Extracellular vesicles (EVs) are membrane-bound particles that vary in size, cargo composition, biogenesis pathways, and delivery mechanisms [1–3] (Figure 1). EVs isolated following high-speed spins of culture supernatant were initially thought to be cellular debris [4] and also referred to as ‘platelet dust’ when isolated from plasma [5]. In the 1970s, vesicles were observed to be secreted by intestinal tuft (caveolated) cells *in situ* [6], and it was later found that secreted gut luminal vesicles had detoxifying activity, reacting with the gut microbiome [7,8]. In 1981, the term exosome was used to refer to membrane vesicles released from cells that contain 5’-nucleotidase and ATPase activity [9]. Later, membrane trafficking studies using reticulocytes showed by electron microscopy that intraluminal vesicles from multivesicular bodies (MVBs or late endosomes) are released from cells; these particles were also referred to as exosomes [10,11].

In the endocytic pathway, early endosomes undergo a series of processing steps leading to the formation of late endosomes, also referred to as Multivesicular Bodies (MVBs) containing multiple intraluminal vesicles (ILVs). MVBs can fuse with either lysosomes for cargo degradation or with the plasma membrane which results in the release of ILVs as small extracellular vesicles (sEVs), traditionally referred to as exosomes. There are multiple mechanisms and factors (ceramide, ESCRT-dependent, and ESCRT-independent) mediating inward budding of intraluminal vesicles (ILVs) that carry cellular protein, lipid, and RNA cargo into MVBs, including endoplasmic reticulum membrane contacts (double headed arrow). Fusion of MVBs with the plasma membrane involves a combination of Rab GTPases and SNARE components with subsequent release of ILVs. Direct budding of vesicles from the plasma membrane also generates a heterogenous array of small and large EVs that are collectively referred to as ectosomes. Filipodium-derived EVs result from membrane budding from filipodia and microvilli. Cells that are undergoing apoptosis generate large apoptotic bodies. Migrasomes are secreted from migrating cells, while oncosomes are derived from cancer cells. Exophers are large vesicles that can contain aggregates and cellular organelles, best characterized in *C. elegans*. By unknown mechanisms (dotted line), small nanoparticles (exomeres and supermeres) are also released from cells that contain protein and RNA cargo.

A functional, physiological role for EVs was first described by Graca Raposo, Clotilde Thery, and colleagues who showed that EVs from B lymphocytes and dendritic cells can display and present antigen on their surface [12,13]. These EVs were capable of activating T-cells in an antigen-specific manner. The demonstration that EVs from B cells could functionally activate T-cells accelerated interest in the EV field which was further increased by findings that EVs contain protein and RNA cargo that can be transferred between donor and recipient cells [14–16]. The field of EV biology has been expanding each year, with total PubMed citations increasing from 282 in 2008 to over 6000 in 2023, fuelled by work to define functional cargo loading and transfer from donor to recipient cells. While much more work is needed to fully define the biogenesis and signalling activity of heterogeneous populations of EVs, they are evolutionarily conserved from bacteria to mammalian cells and are proposed to play key roles in diverse biological processes including immunology, pregnancy, cancer, and developmental biology [17]. All cells release EVs, and an overarching theme of current research into EV biology is that biologically active vesicles participate in cell–cell communication, from near-neighbour interactions to distant cells and organs via the lymphatic system or the bloodstream [1,2,18–21].

Intraluminal vesicles (ILVs) released from MVBs were originally termed exosomes [11], but that name has caused some confusion, in part because they are unrelated to nuclear exosomes which degrade RNA, and because many similar sized vesicles are released from cells that do not originate from MVBs. Accordingly, efforts are underway to better name the different classes of vesicles based on size and biogenesis pathways [3,22,23]. While this may still cause some confusion due to the overlap between different size classes of EVs and their biogenesis pathways, international guidelines are being developed [24]. Broadly, a wide variety of different sized vesicles that are released directly from the plasma membrane are referred to as ectosomes, while release from the endocytic pathway by fusion of MVBs with the plasma membrane is reserved for exosomes (Figure 1). Nevertheless, since these categories cannot be distinguished in bulk purified EV preparations, the field has proposed the use of the terms large EVs (lEVs) and small EVs (sEVs) in the absence of other information about biogenesis pathways. The largest categories of lEVs are apoptotic bodies (500 nm-5um) which originate from apoptotic cells [25], migrasomes (300 nm-3um) which originate from the trail left by migratory cells [26], oncosomes (1-10um) which originate from cancer cells [27], exophers (1-50um) which are large vesicles that can contain organelles released from *C. elegans* neurons [28], and microvesicles (100–1,000 nm) which constitute a heterogeneous group of vesicles that directly bud from the plasma membrane, including a subset of sEVs [29] (Figure 1). The best characterized sublass of sEVs is exosomes (50–150 nm) that are derived from the endocytic system, distinct from sEVs released directly from the plasma membrane. Even among exosomes, there is heterogeneity, as evidenced by the release of amphisomes which are particles that arise by fusion of MVBs with autophagosomes followed by release from the plasma membrane [30].

Besides all of the above lipid bilayer encased particles, increasingly refined purification protocols [31] have identified non-vesicular particles that can also mediate cell–cell communication even penetrating the blood-brain barrier [32–34]. Exomeres and supermeres are small (25–50 nm) non-vesicular particles that contain abundant metabolic components and RNA cargo that can be taken up by recipient cells. These non-membrane bound extracellular particles are not to be confused with non-membrane bound cellular particles such as P-bodies or stress granules [35], but the origin of some cargo content in many different extracellular vesicles can be derived from either P-bodies or stress granules [36–38]. Small RNAs can also be associated with lipoproteins for cell–cell transfer [39,40]. For this review, exosomes will be reserved for small extracellular vesicles (sEVs) released from the endocytic system, while heterogenous vesicles that are directly released from the plasma membrane will be referred to as ectosomes. EVPs (Extracellular Vesicles and Nanoparticles) will be used to refer to all released particles, including membrane-bound vesicles and non-membrane encased nanoparticles (Figure 1).

## Exosome biogenesis

A key event in exosome biogenesis is the formation of Intraluminal Vesicles (ILVs) that bud inward into late endosomes, resulting in multivesicular bodies (MVBs) (Figure 1). While there is heterogeneity in the precise mechanisms that drive different cargoes into ILVs, the canonical pathway is mediated through the action of the Endosomal Sorting Complex Required for Transport (ESCRT) [41,42]. The ESCRT machinery consists of ESCRT-0, I, II, and III complexes which act sequentially to traffic proteins, typically including ubiquitinylated cell surface receptors and other cytoplasmic proteins, to endosomal membranes. Along with phosphoinositides, the Hrs subunit of the ESCRT 0 complex binds to ubiquitinylated cargo and then recruits clathrin, leading to association with endosomal membranes [42]. ESCRT I and II then join and drive inward budding of vesicles that contain ubiquitinylated cargo at membrane microdomains enriched in cholesterol and sphingomyelin [43]. While ESCRT I and II can drive inward budding, ESCRT III, in complex with the ESCRT accessory protein ALIX, participates in fission of the neck of the inward budding vesicle resulting in the formation of ILVs within MVBs [44,45].

Genetic knockdown studies helped identify the function of different ESCRT proteins and associated accessory proteins. Consistent with a key role for early ESCRT components, knockdown of Hrs and another ESCRT I component, TSG101, led to inhibition of EV release [41,46]. However, the knockdown of ESCRT II and III components had little effect on sEV generation, suggesting possible redundancy or specificity for subclasses of sEVs [41]. Additionally, other studies identified variation in the roles of ESCRT complexes on ILV formation [47,48]. As an example, the transmembrane protein syndecan 1 binds to signalling proteins and also interacts with syntenin, which in turn associates with ALIX [47]. Together, these proteins can be associated with endosomal membranes, leading to ILV formation even in the absence of some of the ESCRT components [45]. Similarly, the knockdown of the ESCRT III protein CHMP5 inhibits release of Rab11a-dependent exosomes [49]. Finally, lipids that are abundant in EVs, including cholesterol, ceramide, sphingomyelin, phosphatidylserine, and phosphatidylcholine, may play important roles in the ILV formation and eventual release of MVBs [48,50]. Conversion of sphingomyelin to ceramide by neutral sphingomyelinase 2 is thought to be crucial for ESCRT-independent ILV and sEV formation [48]. Ceramide transfer from ER membranes via CERT may also produce membrane curvature and promote EV biogenesis [51–53]. Beyond their role in ILV formation, variable lipid content in the membranes of EVs might also affect recipient cells upon transfer [50,54–56].

## Ectosome biogenesis

Outward budding of vesicles directly from the plasma membrane can lead to the release of numerous different classes of EVs that have been collectively referred to as ectosomes (Figure 1). The biogenesis of ectosomes is distinct from exosome biogenesis, but for small ectosomes, it can still involve some of the same components [57]. A key difference, particularly for larger EVs, is that instead of intracellular trafficking and fusion of multivesicular bodies with the plasma membrane, rearrangement of the cytoskeleton, lipid asymmetry, and recruited proteins can initiate direct budding of vesicles from the plasma membrane [29,58,59]. Changes in Ca^2+^ concentrations, disassembly of the cytoskeleton, and lipid flippases and floppases can then initiate outward budding followed by membrane scission and release [59,60]. These final steps seem to rely on the ADP-ribosylation factor 6 (ARF6)-mediated actomyosin contraction [61].

## Nanoparticle biogenesis

In the last category of extracellular particle, we will focus on concerns of non-membrane bound nanoparticles, referred to as exomeres and supermeres [32–34]. These particles are small (~30–70 nm) but contain protein and RNA cargo that can be transferred between cells [33]. Their precise biogenesis pathways remain to be elucidated, but their abundance, enrichment in small RNAs and glycolytic enzymes, and their ability to pass the blood–brain barrier expand the impact of cell–cell communication mechanisms by EVPs.

## EVP uptake

While a great deal of effort is currently being expended to understand the loading of protein, lipid, and RNA cargo, how released particles are taken up by recipient cells is less well known [62,63]. EVs contain a variety of transmembrane proteins and receptors allowing some EVs to be taken up by recipient cells using receptor-mediated mechanisms. However, EVs can also be taken up by receptor-independent means via endocytosis, phagocytosis, macropinocytosis, or perhaps direct membrane fusion [62,64–67]. Quantitative analyses suggest that EV fusion and uptake are inefficient and even though some aspects of intracellular EV cargo release have been partially addressed, more work is needed to define mechanisms [64–66,68]. For nanoparticles such as exomeres and supermeres, the lack of membranes may facilitate eventual intracellular release of cargo, but the question remains how or if these particles are targeted to specific cell types. For therapeutic purposes, defining the mechanisms of uptake and cytoplasmic delivery of functional EVP cargo is crucial to enable robust cargo delivery and changes in gene expression in recipient cells.

## EV cargo

Given the heterogeneity in the size and biogenesis of different classes of EVPs, it should not be surprising that there is also great heterogeneity in the protein, RNA and lipid cargo carried within or on EVPs [3,69]. Differences in the concentrations and enrichment of nucleic acids, proteins, and lipids are cell-context dependent and also dependent on the choice of purification protocol [31,70–72]. For the remainder of this review, we will focus on EV protein and RNA cargo, largely leaving nanoparticle contents to other reviews [3].

**Figure 2.**
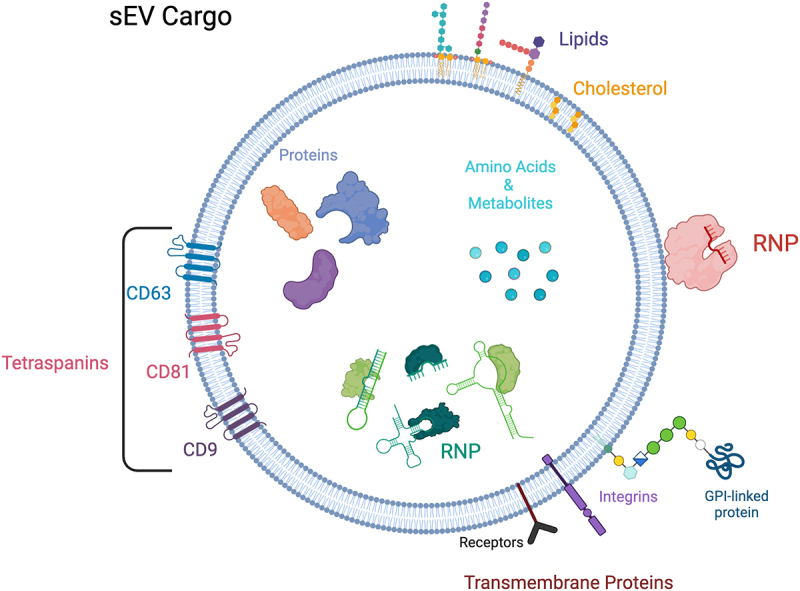
Exosomes.

RNAseq, lipidomic, and proteomic analyses, along with mechanistic studies, all support the notion that export of EV cargo is a regulated event resulting in selective enrichment of specific cargoes [2,19,73]. For sEVs where the biogenesis pathway is better characterized (i.e. exosomes), cargo selection is often associated with proteins that drive vesicle biogenesis leading to enrichment of endosomal and trafficking proteins, along with a variety of cellular proteins (Figure 2). Release of large EVs by direct plasma membrane budding typically results in vesicles whose cargo contents more closely resemble the overall cellular content [74–76]. Here, we will discuss a small subset of EV protein cargo with a more extensive discussion of RNA cargo, particularly miRNA cargo. For a more complete description of the different kinds of EV cargo, the Vesiclepedia website contains published EV cargo contents across an array of vesicles from numerous starting materials (http://microvesicles.org/index.html) [77].

Exosomes and many classes of sEVs are membrane-encased vesicles that contain a variety of associated proteins, lipids, receptors, and internal cargo including metabolites, and RNA-protein complexes (RNPs). Tetraspanins (CD63, CD81 and CD9) are transmembrane protein markers of traditional exosomes.

## Protein cargo

While EVs from diverse sources contain many different proteins, a common set of proteins have been characterized that serve as traditional markers. One family of enriched common EV proteins are tetraspanins, a large family of integral membrane proteins that are found on the plasma membrane as well as in endosomal and lysosomal membranes. They are enriched in small EVs, associating with and regulating internalization of cargo into early endosomes [78]. Among tetraspanins, CD63, CD81 and CD9 are all enriched in sEVs [79] (Figure 2). At the plasma membrane, interactions between tetraspanins and integrins form tetraspanin-enriched microdomains (TEMs) that contain many key EV protein and lipid components [80,81] and which can induce membrane curvature and facilitate vesicle release [78,82,83]. Tetraspanins are also key regulators of cell signalling [84], interacting with and regulating many proteins found in sEVs. These include Epidermal Growth Factor Receptor (EGFR), as well as EGFR ligands and metalloproteinases that process EGFR ligands, leading to autocrine and paracrine signalling [85,86].

Several members of the small GTPase Rab family of proteins function similar to tetraspanins, being involved in vesicle biogenesis and trafficking to membranes [87,88]. Rab11 was the first Rab GTPase shown to be involved in EV secretion [89], but additional work has shown the involvement of several other Rabs including Rab2b, Rab5a, Rab27a and b, and Rab13 [74,87,88]. Different Rabs function at different steps in the process, with some involved in biogenesis and others involved in MVB docking.

There are multiple tetraspanins and Rabs that have been implicated as both EV cargo content and as regulators of cargo content. Assigning specific functions to individual tetaspanins or Rabs can be complicated because of potential genetic redundancy as large protein superfamilies. Broadly, as transmembrane proteins, tetraspanins interact with themselves, other membrane proteins, cytoplasmic proteins, cholesterol and lipids thereby creating a network that can mediate both selection of cargo and EV biogenesis [90]. For the Rab superfamily, individual members localize to a variety of intracellular organelles with different roles throughout the secretory pathway [91]. This could mean that their presence in EVs is simply as passive cargo, but this is counterbalanced by experiments showing roles for specific Rabs in EV biogenesis and secretion [74,87].

## RNA cargo

Because extracellular fluids display abundant ribonuclease activity, extracellular RNAs must be protected from degradation, either in protein complexes, nanoparticles [34,92,93], lipid complexes [40,94], or EVs [15,16,95]. In 2006, cell–cell transfer of mRNAs and proteins was proposed to reprogram haematopoietic precursors [14], and then in 2007, Lotvall and colleagues showed that mRNAs isolated from EVs derived from mouse mast cells could be translated into proteins *in vitro* [16]. Since that time, almost all known subtypes of RNA have been detected across the range of EV classes [76,96–100]. Extensive RNAseq has shown that the great majority of RNA reads within EVs correspond to small (<200 nt) fragments derived from larger RNAs, particularly rRNA and tRNA [70,76]. Mature miRNAs are detectable in EVs and remain the best characterized subclass of RNA in EVs because they are intact and because of the ability to track transfer between cells using standard reporter assays. However, miRNAs often make up only a small percentage of total sEV reads [96] and quantitative analyses indicate that only a small fraction of EVs actually contain intact miRNA [101–103]. This is counterbalanced by the large numbers and heterogeneity of sEVs such that some sEVs may be heavily laden with miRNAs, while others largely lack miRNA [51].

## Regulated miRNA export

Despite quantitative caveats, numerous studies have proposed that miRNA loading into EVs is selective, with distinct subsets of secreted miRNAs when compared to parental cells [38,96,104–108]. The regulatory mechanisms controlling RNA loading into EVs remain an area of intense study, but specific sequence motifs and/or RNA binding proteins have been proposed to mediate regulated, selective export [104,105,108–114]. While there is some overlap in the reported export sequence motifs, no universal sequence or common RNA binding protein has been identified that can unify the various studies. It is possible that when compared to the parental cells, EV RNA profiles could be different because of differential RNA stability or other effects, as opposed to a specific regulatory cascade [115]. These effects could include cell-specific sequence motifs, RNA structures, RNA binding proteins, and RNA-protein condensates [116–121]. A subset of EVs that are enriched in RNA was recently found to be generated at endoplasmic reticulum (ER) membrane contact sites [51]. As some RNAs in RNA-protein granules/condensates are associated with the ER, the specific composition and dynamic state of RNA-containing granules may, in part, define the RNA content of some classes of EV [120]. One possible mechanism that could unify disparate studies regulating RNA export could be a combination of RNA sequence motifs that contain specific RNA-base modifications which are recognized by RNA binding proteins capable of acting as chaperones that deliver RNAs to late endosomes, leading to exosomal accumulation. The same mechanism could also apply to delivery of RNA to non-exosomal and non-vesicular particles that are released by heterogeneous mechanisms. Ultimately, further work is needed to better define the mechanisms governing export and transfer of RNA between cells [70,73,115,122].

## Extracellular RNAs, tumor growth, and progression

Tumours often derive from cells containing mutations that provide a growth advantage. As these cells begin to proliferate, the immediate microenvironment involves crosstalk by EVPs released from both normal and cancer cells. Adjacent normal cells, fibroblasts, endothelial cells, and immune cells often release EVPs that contain miRNA cargo that can inhibit tumour growth. In prostate cancer, normal epithelial cells release *miR-143* that suppresses tumour formation [123]. Similarly, *miR-145* released from stromal cells can suppress tumour formation in pancreatic ductal adenocarcinoma and prostate cancer by inducing apoptosis in recipient cancer cells [124,125]. Counteracting these effects, breast cancer tumour cells secrete *miR-105* which reprograms stromal cells to promote tumour growth [126]. Cancer-associated fibroblasts (CAFs) are one of the major players in the tumour stroma [127]. CAFs are distinct from normal stromal fibroblasts and serve to create a tumour-promoting niche, in part, through remodelling the extracellular matrix. These changes are driven by microenvironment interactions between tumours and CAFs via the release of growth factors, pro-inflammatory molecules and EVPs containing miRNAs that promote tumorigenesis [128]. Several miRNAs have been identified across multiple types of cancer that participate in two-way communication between CAFs and tumour cells that can promote tumour formation including *miR-105, miR-211, miR-9, miR-125b, miR-146, miR-21, miR-1247, miR-142, miR-124, and miR-275* [126,129–131].

Beyond cell–cell communication by EVPs during the earliest stages of cancer, numerous examples of miRNA transfer have been documented that promote tumour growth and progression. Transfer of *miR-146* from leukaemia cells can drive transformation of normal mononuclear cells [132] and uptake of EVPs containing miRNAs that are referred to as oncomirs, notably the *miR-17-92* cluster, can similarly promote oncogenesis in recipient cells [133]. *miR-100* and *miR-125b* are abundant in EVPs released from colorectal cancer cells and can drive tumour growth and promote the epithelial to mesenchymal transition (EMT) [96,134–136]. In lung cancer, the transfer of *miR-21, miR-193* and *miR-201* also promotes EMT [137]. EVPs from CRC cells not only contain abundant *miR-100* and *miR-125b*, they also have abundant levels of EGFR ligands on their surface that can signal through the EGFR to activate downstream signalling cascades and proliferation [85,86,138]. This illustrates that the overall effect of EVPs on recipient cells is likely a combination of miRNA and protein cargo that can induce growth, proliferation, and other aggressive tumour behaviours.

## Extracellular RNAs, motility and invasiveness

As part of a continuum of gene expression and phenotypic changes during which localized tumour cells proliferate and remodel their local environment, the process of metastasis begins when tumour cells undergo gene expression changes that allows them become motile, pass through basement membranes and the extracellular matrix (ECM), invade surrounding tissues, and intravasate into the lymphatic or vascular circulation [139]. The *miR-212/132* cluster plays a role in regulating gene expression in a variety of contexts, but it has also been shown to decrease expression of E-cadherin in oesophageal cancer, leading to increased expression of matrix metalloproteinases, degradation of the ECM, and cancer cell escape from primary tumours [140]. Similarly, *miR-23* is part of a cluster of miRNAs (*miR-23/27/24/2*) that plays multiple roles in cancer, but *miR-23* expression has been shown to promote invasiveness of glioblastoma cells by inducing expression of invasion-related proteins including metalloproteinases and the transcription factors Snail and Slug [141]. The above effects of the *miR-212/132* and *miR-23* clusters are due to cellular expression patterns, but these miRNAs are also abundant in EVs [142,143].

Using spheroid growth conditions, *miR-100* and *miR-125b* were found to promote invasion and motility, due, in part, to the downregulation of cingulin, a protein that links the cytoskeleton to tight junctions and which can promote EMT in CRC cells [134,136]. These miRNAs are abundant in CRC-derived EVs, and the uptake of *miR-100* and *miR-125b* can downregulate cingulin in recipient cells [134]. Likewise, EV-derived *miR-105* targets the tight junction protein ZO-1 to disrupt tight junctions leading to enhanced motility and migration of breast cancer cells [144]. In C2C12 myoblasts and rhabdomyosarcoma cells, *miR-486* is enriched in EVs that act in a paracrine manner to increase proliferation, migration, and invasion in recipient cells [145–147]. In metastatic breast cancer, *miR-21* is enriched in EVs and is required for EMT, at least in part, by targeting Wnt signalling [148]. Broadly, *miR-21* is overexpressed in many cancers [149]. It is highly enriched in serum and EVs from hepatocellular patients, but opposite results have been obtained as to whether it promotes or inhibits migration, invasion and cancer [150–153].

In EVs derived from KRAS mutant CRC cells, *miR-10* is abundant [96]. Increased levels of *miR-10* promote invasion, migration, and metastasis in breast cancer cells by repressing the expression of HOXD10 leading to increased expression of RHOC, matrix metalloproteinase-14, α3 integrin, and urokinase-type plasminogen activator receptor [154]. In contrast, cellular and extracellular levels of *miR-140* from breast cancer cells decrease overall migration and invasiveness, reflecting the reciprocal nature of miRNA exchange between cells [155,156]. Likewise, *miR-320*, which is abundant in EVs from CRC, inhibits invasiveness [157].

## Extracellular RNAs and metastasis

Many EV and cellular miRNAs have been implicated in driving metastasis, with several comprehensive reviews [158–161]. Here, we focus on more recent papers reporting roles for EVs and EV miRNAs in metastasis, focusing first on the metastatic niche. The metastatic niche refers to microenvironment conditions that promote the survival and outgrowth of tumour cells at distant sites [162]. The Lyden lab first published the surprising finding that melanoma exosomes can transfer the receptor tyrosine kinase MET to bone marrow-derived progenitor cells causing reprogramming, proliferation, bone marrow exit, and migration to distant sites, establishing the pre-metastatic niche in organs such as the lungs and bone [163]. They followed up that finding by showing that small EVs from pancreatic cancer cells can similarly contribute to pre-metastatic niche formation in the liver and then expanded their findings to include EVs released from lung, liver, and brain tumour cells capable of enhancing metastasis to distant organs, with surface integrins determining organotropism [164,165]. Their combined data suggest that EVs can alter distant tissues through the delivery of cargo, including proteins that can induce metabolic reprogramming [166,167].

While the Lyden lab papers have not focussed on the miRNA content within EVs that can function to educate tumour cells for future metastasis, several miRNAs have been implicated in multiple stages of tumorigenesis that contribute to metastasis [158–161]. EVs derived from fatty liver can promote metastasis of CRC cells with *miR-103*, *miR-25*, and *miR-92a* being the most enriched [168]. Uptake of EVs containing these miRNAs increases proliferation, migration and invasion when taken up by CRC cells, in part by targeting LATS2 leading to upregulation of YAP signalling. In prostate cancer, *miR-150* was found to be expressed 30-fold higher in metastatic lymph nodes compared to tumour tissue [169]. In breast cancer, *miR-200*, which is known to promote EMT [170], is enriched in the serum of patients with metastatic disease and the transfer of EVs containing *miR-200* can induce EMT and promote metastasis in nonmetastatic cells [171]. This is consistent with a role for the *miR-200* family (*miR-200a, miR-200b, miR-429, miR-141, miR-200c*) in promoting metastasis in hepatocellular carcinoma, brain, and ovarian cancer [172–174].

## Extracellular RNAs and immune evasion

A key early paper on cell–cell communication mediated by sEVs was the discovery that B cells and dendritic cells release sEVs that display peptide-MHC class II complexes on their surface that are capable of activating antigen-specific T cell responses [12,13]. Since then, modulation of both innate and adaptive immunity has been one of the better characterized activities of EVs [18,175]. For innate immunity, a variety of cytokines and signalling molecules are carried on or within EVs that can modulate inflammatory responses, both pro- and anti-inflammatory [176]. For adaptive immunity and related to antigen presentation, EVs can play a role in immune synapse communication between antigen presenting cells and lymphocytes [177], particularly by the transfer of miRNA [95]. Beyond priming the immune system, EVs also play a role in cancer cell immune evasion. Immune checkpoint inhibition of Programmed Cell Death Protein 1 (PD-1) or its ligand 1 (PD-L1) has proven to be an effective immunotherapy for a variety of cancers [178,179]. PD-L1 is a membrane-bound protein whose expression is often upregulated in cancer and which binds to the PD-1 receptor on T-cells leading to downregulation or inactivation of T cells allowing for tumour escape from the immune system [180]. Analysis of EVs released from different tumour cells identified the presence of membrane-bound PD-L1 [181–183]. EVs laden with PD-L1 can bind to PD-1 on cytotoxic T cells leading to anti-tumour immunity [181,184,185]. In chronic lymphocytic leukaemia, tumour-derived EVs can also modulate PD-L1 expression in monocytes, creating a pro-inflammatory tumour microenvironment that facilitates immune escape [179].

EVs also play a role in innate immunity through the action of EV RNA. A hallmark of innate immunity is activation of inflammatory responses mediated by interferons, interferon stimulated genes, and activation of NFkB [186]. The innate immune system encodes Pattern Recognition Receptors that recognize pathogen encoded molecules (Pathogen-Associated Molecular Patterns; PAMPs) to protect the host by initiating immune recruitment. Recognition of foreign nucleic acid is a key component of the innate immune system through the action of RNA sensors. Among these, a subset of the Toll-like Receptors (TLR 7/8) are found on endocytic membranes and can recognize RNAs to activate inflammatory signalling [187–189]. While it remains a question as to how cells distinguish between self and foreign miRNAs in endocytic compartments, foreign small RNAs carried by LDL particles can drive inflammatory responses in macrophages as part of atherosclerosis [39]. Also, Y RNAs in EVs released by tumour cells in chronic lymphocytic leukaemia can be taken up by macrophages, leading to TLR 7 signalling and ultimately increased expression of PD-L1 and a pro-tumorigenic environment [179]. Lastly, *miR-29a* and *miR-21* are released by lung tumour cells and can bind to TLR8 and promote metastasis as part of an inflammatory response [190]. For all of these RNAs, whether they are bound to the external surface of EVs or are somehow released from EVs to interact with endosomal TLR 7/8 remains to be determined.

For EV miRNA-mediated immune evasion by tumour cells, it was discovered that nasopharyngeal carcinoma cells release EVs enriched in *miR-24* that inhibit T cell proliferation and differentiation and induce regulatory T cells (Tregs) that altogether suppress T cell responses and allow immune evasion [191]. Similarly, pancreatic cancer cells release EVs enriched in *miR-203* and *miR-212* that can be transferred to dendritic cells to suppress immune responses and allow tumour cell escape [192,193]. Finally, M2 macrophages (often called tumour-associated macrophages) can assist tumour growth and metastasis by promoting angiogenesis, secretion of metalloproteinases that target the ECM, and suppression of immune responses to tumour cells [194]. Pancreatic and ovarian tumour-derived exosomes enriched in *miR-222, miR-301*, and *miR-940* can promote M2 macrophage polarization and promote metastasis [195–197].

## Extracellular RNAs and cancer drug resistance

Besides immune escape, several reports have shown that EVPs can transfer protein and RNA cargo to mediate drug resistance during cancer treatment [198–200]. Cetuximab and panitumumab are two monoclonal antibodies that target the Epidermal Growth Factor Receptor to block signalling through the Ras/Raf/MAPK pathway [201]. Resistance to these monoclonal antibodies is common, often due to mutations in EGFR [202]. In contrast, we derived a CRC cell line that does not contain EGFR mutations but is nevertheless resistant to cetuximab [203]. These cells dramatically overexpress the MIR100HG locus leading to large fold-changes in the *MIR100HG* lncRNA and two miRNAs (*miR-100* and *miR-125b*). These miRNAs are enriched in EVs from CRC cells and we have shown that cetuximab resistance can spread via EVPs [34,204,205], in part due to activation of Wnt signalling [203,206,207]. Additional work has shown that *MIR100HG, miR-100* and *miR-125b* contribute to tumour growth and invasiveness [134], but overall cetuximab resistance is driven by contributions from multiple factors and not just *miR-100* or *miR-125b*. In contrast, EV transfer of *miR-100* can increase cisplatin resistance in lung cancer [208] and EVs carrying *miR-125b* can increase resistance to rituximab in B-cell lymphoma [209].

Beyond *miR-100* and *miR-125b*, numerous publications have demonstrated that EVP-mediated RNA and miRNA transfer can drive cancer drug resistance in many different cancers (Table 1). Sunitinib resistance has been shown to be promoted by the expression of *lncARSR* that acts to inhibit *miR-34* and *miR-449* in renal cancer [210]. In ovarian cancer, EV transfer of *miR-429* can promote resistance to cisplatin, paclitaxel, and cytoxan, while *miR-21* can confer resistance to paclitaxel [211,212]. In pancreatic cancer, EV delivery of *miR-365*, *miR-210*, or *miR-106b* can all induce resistance to gemcitabine [215,216,219]. In CRC, EV secretion of *miR-208b* by oxaliplatin-resistant cancer cells can be taken up by Tregs to promote their expansion, resulting in immune escape and enhanced tumour growth [220]. In glioblastoma, EV transfer of *miR-25* can promote resistance to temozolomide, while in breast cancer, EVs containing *miR-181b* can transfer doxorubicin resistance [217,218], and transfer of *miR-106a* and *miR-548* can transfer cisplatin resistance to nasopharyngeal cancer [213,214].

**Table 1.** miRNAs involved in cancer drug resistance. miRNAs associated with various types of cancer drug resistance are as indicated.

Cancer Type	Drug Resistance	RNA	Reference
Renal Cancer	Sunitinib	*LncARSR targets miR-34 and miR-499 to promote resistance*	[210]
Lung Cancer	Cisplatin	*miR-100*	[208]
Ovarian Cancer	Paclitaxel	*miR-21*	[211]
Ovarian Cancer	Cisplatin	*miR-429*	[212]
Ovarian Cancer	Cisplatin	*miR-548aq*	[213]
Nasopharyngeal Carcinoma	Cisplatin	*miR-106a*	[214]
Pancreatic Cancer	Gemcitabine	*miR-106b*	[215]
Pancreatic Cancer	Gemcitabine	*miR-210*	[216]
Breast Cancer	Doxorubicin	*miR-181b*	[217]
Glioblastoma	Temozolomide	*miR-25*	[218]
Pancreatic Adenocarcinoma	Gemcitabine	*miR-365*	[219]
Colorectal Cancer	Cetuximab	*MIR100HG, miR-100 and miR-125b*	[203,207]

## Summary and future directions

EVs and nanoparticles are now recognized as novel mediators of cell–cell communication, especially in the context of cancer. All cells release a heterogenous mix of different sized EVPs and ongoing work is devoted to precisely defining the mechanisms controlling their biogenesis and release. The cargo content of EVPs, especially sEVs, is often enriched in specific protein, lipid or RNA cargo, suggesting regulation of cargo export by donor cells. How EVs are taken up by recipient cells for cytoplasmic delivery of cargo remains a key area of needed research, especially better quantification of the effects of EVPs on gene expression patterns or other phenotypic changes in recipient cells.

### Biomarkers and diagnostics

The presence of tumour miRNAs in EVs derived from various cancers raises the possibility of developing non-invasive biomarkers, not only for diagnoses but also to monitor the effectiveness of chemotherapy or other treatments [221]. Glioblastoma, which is aggressive, difficult to treat, and difficult to monitor, is a good example of a disease where EV-enriched proteins or RNAs in the bloodstream or urine would provide a tool to more precisely craft and follow treatment regimens [222]. Another example comes from the Lyden lab which has found that tumour EVs enriched in palmitic acid can induce fatty liver formation [166]. This provides two potential diagnostic/biomarker possibilities. The first would be the recognition that fatty liver disease may mean the presence of extrahepatic tumours, the second is that palmitic acid enriched EVs could serve as a biomarker for cancer. These are just two examples, but similar markers could be developed for many of the miRNAs discussed above.

### Therapeutics

**Figure 3.**
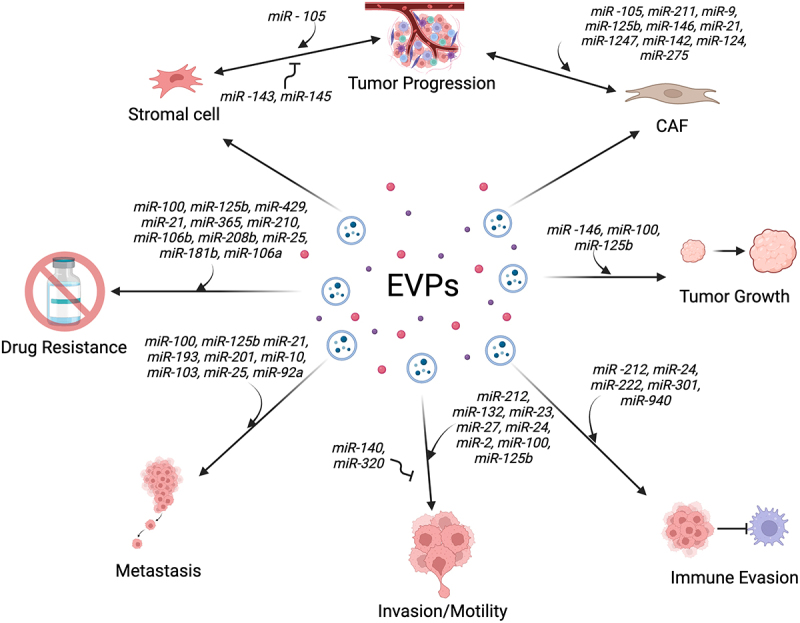
Extracellular vesicles and nanoparticles (EVPs) in tumor progression, invasion, motility, metastasis, immune evasion, and cancer drug resistance.

For cancer, EVPs play an important role in all aspects of oncogenesis through local and distant tumour microenvironment interactions by the delivery of protein and RNA cargo (Figure 3). Determination of mechanisms that regulate EVP-mediated communication has the potential to uncover new therapeutic targets for cancer treatment. Related to fatty liver, the blockage of EV secretion by ablation of Rab27a was able to restore normal liver function [166]. Because all cells release EVs, the ability to selectively block EV secretion could produce unanticipated complications, but efforts are now underway to identify potent inhibitors of EV release with the ongoing challenge to determine how to target such inhibitors to specific cells [223].

EVPs constitute a novel form of cell–cell communication by the transfer of protein and RNA cargo that can impact all stages of oncogenesis. Numerous miRNAs are contained in EVPs that contribute to the indicated steps across many different cancers.

Another obvious potential therapeutic benefit of EVs would be to use them as delivery vehicles for cellular components such as miRNAs [67] or other small molecules. Synthetic nanoparticles are being developed for therapeutic delivery, but it is possible that using modified EVs could be more effective with potential reduced immune clearance and toxicity [224]. The challenge remains to develop tools to target EVs to specific cell types, but the potential for therapeutic approaches to improve human health remains a tantalizing possibility. As the field defines specific biogenesis cascades and regulatory mechanisms, the ability to use EVPs for therapeutic and diagnostic purposes, not to mention better understanding of basic cellular biology, suggests even further excitement in this burgeoning new field.
